# Thromboembolic prophylaxis in neurosurgical practice: a systematic review

**DOI:** 10.1007/s00701-023-05792-3

**Published:** 2023-10-05

**Authors:** Zhaoyuan Zhang, Husule Cai, Carmen L. A. Vleggeert-Lankamp

**Affiliations:** 1 Department of Neurosurgery, Leiden University Medical Centre, P.O. Box 9600, 2300 RC Leiden, The Netherlands; 2 Spaarne Hospital, Hoofddorp, Haarlem, The Netherlands

**Keywords:** Venous thromboembolism, Neurosurgery, Postoperative complications, Prevention and control, Systematic review

## Abstract

**Background:**

In neurosurgical patients, the risk of developing venous thromboembolism (VTE) is high due to the relatively long duration of surgical interventions, usually long immobilization time after surgery, and possible neurological deficits which can negatively influence mobility. In neurosurgical clinical practice, there is lack of consensus on optimal prophylaxis against VTE, mechanical or pharmacological.

**Objective:**

To systematically review available literature on the incidence of VTE in neurosurgical interventions and to establish an optimum prevention strategy.

**Methods:**

A literature search was performed in PubMed, Embase, Web of Science, Cochrane Library, and EmCare, based on a sensitive search string combination. Studies were selected by predefined selection criteria, and risk of bias was assessed by Newcastle–Ottawa Quality Assessment Scale and Cochrane risk of bias.

**Results:**

Twenty-five studies were included, half of which had low risk of bias (21 case series, 3 comparative studies, 1 RCT). VTE was substantially higher if the evaluation was done by duplex ultrasound (DUS), or another systematic screening method, in comparison to clinical evaluation (clin). Without prophylaxis DVT, incidence varied from 4 (clin) to 10% (DUS), studies providing low molecular weight heparin (LMWH) reported an incidence of 2 (clin) to 31% (DUS), providing LMWH and compression stockings (CS) reported an incidence of 6.4% (clin) to 29.8% (DUS), and providing LMWH and intermittent pneumatic compression devices (IPC) reported an incidence of 3 (clin) to 22.3% (DUS). Due to a lack of data, VTE incidence could not meaningfully be compared between patients with intracranial and spine surgery. The reported incidence of pulmonary embolism (PE) was 0 to 7.9%.

**Conclusion:**

Low molecular weight heparin, compression stockings, and intermittent pneumatic compression devices were all evaluated to give reduction in VTE, but data were too widely varying to establish an optimum prevention strategy. Systematic screening for DVT reveals much higher incidence percentages in comparison to screening solely on clinical grounds and is recommended in follow-up of neurosurgical procedures with an increased risk for DVT development in order to prevent occurrence of PE.

## Introduction 

Thromboembolic prophylaxis is a crucial aspect of patient care in neurosurgical practice. Neurosurgical patients are at high risk for thromboembolic events, including deep vein thrombosis (DVT), pulmonary embolism (PE), and stroke. The risk of venous thromboembolic events (VTE) in neurosurgical patients is multifactorial, with several factors contributing to the development of these events [23]. One of the major contributors is the often occurring immobility during and bed rest after (lengthy) surgery, which can lead to venous stasis and impaired blood flow [27]. Other factors such as the use of vasopressors, dehydration, and motor deficits pre- and post-surgery also increase the risk of thromboembolic events [17, 23]. Finally, another factor that may lead to an increased risk of a thromboembolic event is a state of hypercoagulability, induced by the presence of malignant tumors or subarachnoid hemorrhage [44].

The clinically relevant symptoms associated with DVT are warmth, swelling, pain and redness of the leg, but the majority of VTE cases are asymptomatic [11, 17]. Meanwhile, the asymptomatic VTE localized in deep lower extremity veins may progress to symptomatic VTE [22, 28, 32, 37]. As for clinical examination, DVT can be confirmed by Doppler ultrasound (DUS) or venography [23, 45], and PE confirmed by computed tomography (CT) of the chest or angiography (CTA) [23, 45].

The CHEST Guidelines recommend that every hospital develops a formal strategy that addresses the prevention of VTE [15]. Thromboembolic prophylaxis includes pharmacological measures with usually low molecular weight heparin (LMWH) and non-pharmacological measures as early mobilization and physical therapy, compression stockings, and intermittent pneumatic compression (IPC) devices [15]. For “patients undergoing major neurosurgery,” the CHEST Guidelines recommend “optimal use of mechanical methods of thromboprophylaxis” with the acceptable alternative of LMWH. For this type of patients with a “particular high thrombosis risk,” it is recommended to combine mechanical prophylaxis with LMWH. In patients undergoing neurosurgery with high bleeding risk, the mechanical method is recommended to substitute LMWH [15]. Definitions are however not further specified.

Lack of knowledge on prophylaxis of thromboembolic events in neurosurgical clinical practice leads to absence of consensus on the choice of prophylaxis for VTE in the Netherlands. A wide diversity in choice (IPC, compression stockings, heparin, LMWH) and timing (preoperative, postoperative) of prophylactic measures was shown in an evaluation of the use of VTE prophylaxis in all seven university neurosurgical clinics in the Netherlands [26].

Previously, in 2012, we performed an extensive literature search on the incidence of thromboembolic events in patients undergoing spinal or intracranial neurosurgical procedures and summarized our results in a systematic review [26]. It was concluded that intracranial surgical patients were more at risk to develop a VTE compared to spinal surgery patients, that the use of antithrombotic prophylaxis in neurosurgical interventions lowers the VTE incidence from 30 to about 1.5 to 6%, that a twofold higher VTE rate was demonstrated in patients systematically screened for DVT in comparison to those solely clinically screened, and that subclinical DVT was described to be associated with the incidence of PE. However, large heterogeneity with respect to diagnostic methods for VTE events and variable antithrombotic prophylaxis prevented us from drawing firm conclusions on optimal treatment strategy. Now, 10 years later, we deemed it useful to perform an update of this review with the purpose of finding more definite answers to optimizing anti-thrombotic treatment strategy in neurosurgical patients.

## Methods

### Literature search strategy

A systematic review of the literature was performed by following the Preferred Reporting Items for Systematic Reviews and Meta-Analysis (PRISMA) guidelines [31]. A comprehensive search strategy in PubMed, Embase, and Web of Science was executed to examine the incidence of VTE in the perioperative care in neurosurgery. The search strategy is shown in Appendix 1. Dates of the search queries included articles from October 2012 up to and including January 2022. This time course was chosen to expand beyond the literature search of our previous systematic review [26].

### Eligibility criteria

Selection of articles was independently performed by two reviewers (ZZ and CV-L). The inclusion criteria were in line with the aforementioned review. Articles were considered eligible for inclusion if it concerned patients that underwent a neurosurgical intervention, if VTE was systematically evaluated and the method with which DVT and/or PE was diagnosed was clearly indicated. Both the nature of the intervention (intracranial or spinal intervention) and the anti-thrombotic prophylaxis (none, mechanical, chemical, or a combination) had to be clearly described. Case studies with a minimum of ten patients, cohort studies, and randomized controlled trials could be included; systematic reviews and meta-analyses were excluded.

After screening for eligibility according to the inclusion and exclusion criteria, the articles were further analyzed for relevance to determine the final article selection. Consensus about the selection was reached in open discussion.

### Quality assessment

Three investigators (ZZ, HC, and CV-L) independently performed a risk of bias analysis by assessing the included observational cohort studies according to an adjusted Newcastle–Ottawa Scale (NOS) (Appendix 2) [46]. Additionally, the Cochrane risk of bias tool (Appendix 3) was used for comparative studies and randomized controlled studies. Any disagreements between the three investigators were resolved. Maximum scores were 4 for selection, 1 for comparability, and 4 for outcome assessment. The risk of bias was then ranked as high (≤ 2 points), moderate (3–4 points), or low (5 or more points) depending on the overall score. A score of 6 points or more (out of a maximum of 12 points) on the Cochrane risk of bias tool was defined as a low risk of bias.

### Diagnosis of venous thromboembolism

DVT and PE can be diagnosed on clinical grounds. Symptoms of DVT include pain, swelling, redness, and warmth of the skin of the leg (usually the calf). The presence of symptoms can lead to a clinical diagnosis of DVT, which thereafter may or may not be confirmed with objective imaging or measurements. Symptoms of PE are pain in the thoracic area and dyspnoea. The clinical diagnosis of PE is generally followed by objective imaging evaluation due to its essential need for treatment.

DVT can also be systematically evaluated using a more objective screening method to evaluate the presence of DVT. This means that all participants in a study, regardless of whether or not they experience symptoms, will be evaluated using an objective screening method. The most common screening method to screen for DVT is by postoperative duplex ultrasound of the legs. Another screening method is the evaluation of D-dimer in a blood sample [16, 18, 19, 21, 30, 36, 41]. To further evaluate PE, computed tomography (CT) of the chest [3, 7, 18, 40, 42, 43] or angiography (CTA) [1, 6, 10, 20, 38] is the most commonly performed evaluation method; other evaluation methods are a ventilation–perfusion scan (VQ scan) [6, 39, 43] or a the pulmonary arteriogram [39].

### Data extraction and analysis

Data was extracted from each article by one investigator (ZZ) and reviewed by a second investigator (CV-L). Disagreements between the reviewers were resolved by consensus. The following data were extracted from the included studies: study design, sample size, type of neurosurgical intervention (intracranial or spinal), type of antithrombotic prophylaxis, method of DVT diagnosis, and method of PE diagnosis, and, if mentioned, incidence of hemorrhage. The primary outcome parameter assessed was the occurrence of DVT and/or PE. If risk factors for VTE were evaluated, odds ratios or hazard ratios for each risk factor that was analyzed in a multivariate analysis were collected.

In order to calculate the average incidence of VTE in neurosurgical patients, data were pooled.

## Results

### Search and selection results

**Fig. 1:**
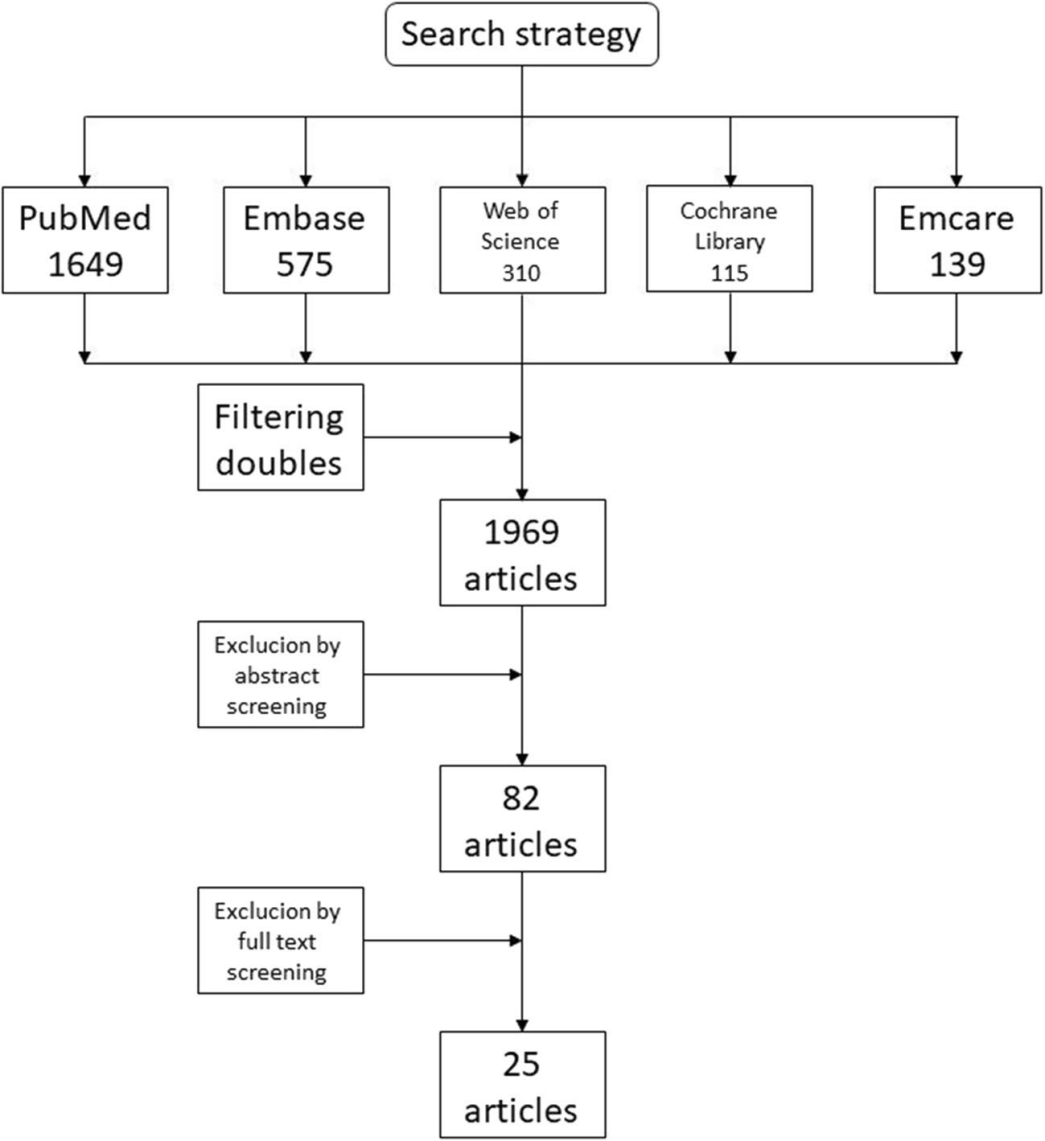
Flow diagram depicting the study selection process

The search yielded 1969 unique references. After screening titles and abstracts, 82 articles were subjected to full text review (Fig. 1). A total of 51 studies were excluded after full text review due to the absence of specific information about the diagnostic method for DVT and only reporting bleeding complications. Twenty-five articles were subjected to quality assessment. Due to insufficient data, pooling of the data was not deemed meaningful, and only a descriptive analysis was performed.

### Study characteristics

Of the 25 included studies, 1 was a randomized controlled trial [35], 3 were comparative studies [8, 13, 14], and 21 were case series (Tables 1 and 2). Twelve studies reported patients subjected to intracranial surgery. Two studies reported patients undergoing spinal surgery. Eleven studies reported patients both subjected to intracranial and spinal surgery (intracranial/spinal). Eight studies diagnosed DVT on clinical grounds [7, 10, 13, 14, 38, 41–43], and sixteen studies diagnosed DVT based on systematic screening methods, using D-dimer [16, 18, 19, 21, 30, 36], DUS [1–3, 6, 8, 16, 18–21, 25, 30, 34–36, 47], or CT [47]. One study evaluated both on clinical grounds and performed routine screening (DUS/CT), with the purpose of comparing the two methods [40]. Low molecular weight heparin (LMWH), unfractionated heparin, intermittent pneumatic compression (IPC) devices, compression stockings (CS) peri- and/or postoperatively, or a combination of these were described as prophylaxis methods (Table 3).

**Table 1:** Risk of bias (case series)

Author, year	Type of neurosurgical intervention	Design of study	Total risk of bias	Newcastle–Ottawa Assessment Scale		
				Selection	Comparability	Outcome
Chaichana, 2013 [7]	Intracranial	Case series	High	■■□□	□	■■□□
Smith, 2014 [43]	Intracranial	Case series	High	■■□□	□	■□□□
Hoefnagel, 2014 [18]	Intracranial	Case series	High	■■■□	□	□□□□
Daley, 2015 [10]	I**ntracranial**	C**ase series**	**Low**	**■■■□**	**□**	**■■□□**
Sjåvik, 2016 [42]	Intracranial	Case series	High	■■□□	□	■■□□
Nakano, 2018 [30]	I**ntracranial**	C**ase series**	**Low**	**■■■■**	**□**	**■■■□**
Rinaldo, 2019 [38]	Intracranial	Case series	High	■■□□	□	■■□□
Ali, 2019 [3]	Intracranial	Case series	High	■□□□	□	■□□□
Kaewborisutsakul, 2020 [20]	I**ntracranial**	C**ase series**	**Low**	**■■■■**	**□**	**■■■□**
Shi, 2020 [41]	I**ntracranial**	C**ase series**	**Low**	**■■■□**	**□**	**■■□□**
Al-Dujaili, 2012 [2]	**Spinal**	C**ase series**	**Low**	**■■■□**	**□**	**■■■■**
Ikeda, 2017 [19]	**Spinal**	C**ase series**	**Low**	**■■■□**	**□**	**■■■■**
Patel, 2013 [34]	I**ntracranial/spinal**	C**ase series**	**Low**	**■■■■**	**□**	**■■■□**
Guo, 2015 [16]	I**ntracranial/spinal**	C**ase series**	**Low**	**■■■■**	**□**	**■■■■**
Carrabba, 2018 [6]	I**ntracranial/spinal**	C**ase series**	**Low**	**■■■□**	**□**	**■■■□**
Samuel, 2019 [40]	I**ntracranial/spinal**	C**ase series**	**Low**	**■■■□**	**□**	**■■■■**
Rethinasamy, 2019[36]	Intracranial/spinal	Case series	High	■■□□	□	■■□□
Agarwal, 2019 [1]	I**ntracranial/spinal**	C**ase series**	**Low**	**■■■□**	**■**	**■■■□**
Yun, 2019 [47]	Intracranial/spinal	Case series	High	■■□□	□	■■□□
Karsy, 2020 [21]	I**ntracranial/spinal**	C**ase series**	**Low**	**■■■■**	**□**	**■■■□**
Li, 2020 [25]	I**ntracranial/spinal**	C**ase series**	**Low**	**■■■■**	**□**	**■■■□**

**Table 2:** Risk of bias (RCT and comparative studies)

Author, year	Type of neurosurgic al intervention	Design of study	Total risk of bias	Cochrane risk of bias tool	Newcastle–Ottawa Assessment Scale		
					Selection	Comparability	Outcome
Prell, 2018 [35]	**Intracranial**	**RCT**	**Low**	■■■■■■□□□□□□	■■■■	■	■■■■
Eisenring, 2013 [14]	Intracranial/spinal	Comparative studies	High	■■■■□□□□□□□□	■□□□	■	■■□□
Chibbaro, 2018 [8]	Intracranial/spinal	Comparative studies	High	■■■□□□□□□□□□	■■■□	□	■■□□
Ebeling, 2018 [13]	Intracranial	Comparative studies	High	■■■■□□□□□□□□	■□□□	□	■□□□

**Table 3:** Incidence of VTE categorized by type of operations

Author, Year	Type of operations	Study design	Prophylaxis	Sample size	Incidence of VTE (%)	Incidence of DVT (%)	Incidence of PE (%)	Diagnosis of VTE/DVT
Daley, 2015 [10]	intracranial	case series	no prophylaxis	226	4.0	3.0	1.0	clinical
			LMWH	45	2.0	2.0	0.0	
Hoefnagel, 2014 [18]	intracranial	case series	LMWH	581	7.9	3.4	4.5	D-Dimer, DUS
Sjåvik, 2016 [42]	intracranial	case series	LMWH routinely	626	3.8			clinical
		case series	LMWH as needed	353	3.1			
Smith, 2014 [43]	intracranial	case series	2 heparin, 23LMWH	336	15.8			clinical
Ali, 2019 [3]	intracranial	case series	4 heparin 1-4 days, 4 LMWH 1-2 days, the other no	387	1.3	0.5	0.8	DUS
Rinaldo, 2019 [38]	intracranial	case series	LMWH+IPC	1622	3.0	2.3	0.9	clinical
Chaichana, 2013 [7]	intracranial	case series	IPC + heparin	1277	3.1	2.8	0.3	clinical
Ebeling, 2018 [13]	intracranial	comparative study	CS+LMWH	78	6.4	3.8	2.6	clinical
			CS+LMWH+IPC	75	9.3	8.0	1.3	
Prell, 2018 [35]	intracranial	RCT	CS+LMWH	53	26.4			DUS
			CS+LMWH+IPC	41	7.3			
Nakano, 2018 [30]	intracranial	case series	CS+IPC	61	21.3			DUS, D-dimer
Shi, 2020 [41]	intracranial	case series	CS+IPC	1670	13.4	12.9	0.5	clinical
Kaewborisutsakul, 2020 [20]	intracranial	case series	IPC	177	10.2	8.5	3.4	DUS
Al-Dujaili, 2012 [2]	spinal	case series	LMWH+CS	158	0.6	0.6		DUS
Ikeda, 2017 [19]	spinal	case series	CS+IPC (perioperative)	194	29.4	29.4		DUS, D-dimer
Rethinasamy, 2019 [36]	intracranial/spinal	case series	no prophylaxis	320	10.3	10.3		D-dimer (>2mg/L), DUS
Guo, 2015 [16]	intracranial/spinal	case series	LMWH	196	31.1	31.1		DUS, D-dimer
Agarwal, 2019 [1]	intracranial/spinal	case series	LMWH+IPC	11436	0.6	0.2	0.4	DUS
Patel, 2013 [34]	intracranial/spinal	case series	IPC + heparin	1277	3.1	2.8	0.3	DUS
Carrabba, 2018 [6]	intracranial/spinal	case series	LMWH+CS	275	29.8		2.9	DUS
Samuel, 2019 [40]	intracranial/spinal	case series	pharmacologic and mechanical prophylaxis (standard screening)	104	10.6	10.6		clinical
		case series	pharmacologic and mechanical prophylaxis (routine DUS screening)	53	9.4	9.4		DUS
Karsy, 2020 [21]	intracranial/spinal	case series	IPC+Heparin/LMWH	1918	22.3	22.3		DUS, D-dimer
Li, 2020 [25]	intracranial/spinal	case series	mechanical and pharmacologic prophylaxis	204	30.9	30.9		DUS
Yun, 2019 [47]	intracranial/spinal	case series	pharmacologic with/without mechanical prophylaxis	13913	1.8			DUS/CT
Eisenring, 2013 [14]	intracranial/spinal	comparative study	CS+LMWH	482	12.7	4.8	7.9	clinical
			IPC+LMWH	242	7.1	4.6	2.5	
Chibbaro, 2018 [8]	intracranial/spinal	comparative study	CS+LMWH	3169	4.2	3.0	1.2	DUS
			CS+LMWH+IPC	3818	1.5	1.3	0.2	

*VTE* venous thromboembolism; *DVT* deep venous thrombosis; *PE* pulmonary embolism; *CS* compression stockings; *IPC* intermittent pneumatic compression; *LMWH* Low Molecular weight heparin, *DUS* Duplex ultrasound, *CT* computed tomography

### Assessment of risk of bias

Thirteen out of the 21 case series were rated to have a low risk of bias, having a score of 5/9 points on the Newcastle–Ottawa Quality Assessment Scale (Table 1), and one of the comparative studies (the RCT) was assessed to have a low risk of bias. The other three comparative studies were all scored to have a high risk of bias (Table 2).

### VTE incidence related to prophylaxis strategy in intracranial surgery

The incidence of VTE in patients after intracranial surgery ranged from 1.3 to 26.4% (Table 3). Only one study yielded data for absence of prophylaxis for VTE and reported an incidence of 4.0%; diagnosis was made on clinical grounds without systematic screening of all patients. In patients receiving pharmacological prophylaxis (LMWH or heparin), the incidence of VTE diagnosis ranged from 1.3 to 7.9% (using systematic screening methods) and 2.0 to 3.8% if diagnosis was based on clinical evaluation. A deviating outcome was reported by Smith [43], evaluating VTE on clinical grounds and demonstrating an incidence of 15.8%. Only one article studied the incidence of DVT in patients being subjected to IPC as single prevention method and reported an incidence of 10.2% (screening by DUS). In the two articles describing patients who received both IPC and CS, the incidence was 13.4% evaluated on clinical grounds and 21.3% if DVT was diagnosed based on systematic screening. In patients in whom the administration of pharmacologic prophylaxis was combined with CS and/or IPC, the VTE rate was ranging from 3.1 to 6.4% based on clinical evaluation and from 7.3 to 26.4% based on systematic screening methods.

### VTE incidence related to prophylaxis strategy in spinal surgery

One article studied the incidence of VTE using both IPC and CS as prophylactic method and reported a 29.4% incidence of VTE using DUS as systematic screening method. In patients in whom the administration of pharmacologic prophylaxis was combined with CS and/or IPC, the VTE rate was 0.6% based on systematic screening methods.

### VTE incidence related to prophylaxis strategy in intracranial/spinal surgery

The incidence of VTE in patients after intracranial/spinal surgery ranged from 0.6 to 31.1% (Table 3). If no prophylaxis for VTE was provided, incidence was reported to be 10.3%, using systematic screening. In patients receiving pharmacologic prophylaxis such as LMWH and heparin, a 31.1% incidence of VTE was reported using systematic screening. In patients in whom the administration of pharmacologic prophylaxis combined with CS and/or IPC, the VTE rate was 7.1 to 12.7% based on clinical evaluation and 0.6 to 30.9% based on systematic screening methods.

### VTE incidence categorized by prophylaxis strategy

VTE incidence was categorized by prophylaxis strategy in Table 4. If no prophylaxis for VTE was provided, VTE was reported to occur in 4.0 (clinical evaluation) to 10.3% (systemic evaluation) of patients. In patients receiving pharmacologic prophylaxis such as LMWH and heparin, a 1.3 to 31.1% incidence of VTE was reported. In patients who received IPC with CS, the incidence was 13.4 to 29.4%, while the incident in patients who received IPC alone was 10.2%. In patients in whom the administration of pharmacologic prophylaxis combined with IPC and/or CS, the VTE rate was 0.6 to 22.3%, and in patients whom the administration of pharmacologic prophylaxis combined with CS (without IPC), the VTE rate was 0.6 to 29.8%.

**Table 4:** Incidence of VTE categorized by prophylaxis strategy

Author, Year	Type of operations	Study design	Prophylaxis	Sample size	Incidence of VTE (%)	Incidence of DVT (%)	Incidence of PE (%)	Diagnosis of VTE/DVT
Daley, 2015 [10]	intracranial	case series	no prophylaxis	226	4.0	3.0	1.0	clinical
Rethinasamy, 2019 [36]	intracranial/spinal	case series	no prophylaxis	320	10.3	10.3		D-dimer (>2mg/L), DUS
Daley, 2015 [10]	intracranial	case series	LMWH	45	2.0	2.0	0.0	clinical
Hoefnagel, 2014 [18]	intracranial	case series	LMWH	581	7.9	3.4	4.5	D-Dimer, DUS
Sjåvik, 2016 [42]	intracranial	case series	LMWH routinely	626	3.8			clinical
		case series	LMWH as needed	353	3.1			
Smith, 2014 [43]	intracranial	case series	2 heparin, 23LMWH	336	15.8			clinical
Ali, 2019 [3]	intracranial	case series	4 heparin 1-4 days, 4 LMWH 1-2 days, the other no	387	1.3	0.5	0.8	DUS
Guo, 2015 [16]	intracranial/spinal	case series	LMWH	196	31.1	31.1		DUS, D-dimer
Nakano, 2018 [30]	intracranial	case series	CS+IPC	61	21.3			DUS, D-dimer
Shi, 2020 [41]	intracranial	case series	CS+IPC	1670	13.4	12.9	0.5	clinical
Ikeda, 2017 [19]	spinal	case series	CS+IPC (perioperative)	194	29.4	29.4		DUS, D-dimer
Kaewborisutsakul, 2020 [20]	intracranial	case series	IPC	177	10.2	8.5	3.4	DUS
Rinaldo, 2019 [38]	intracranial	case series	LMWH+IPC	1622	3.0	2.3	0.9	clinical
Chaichana, 2013 [7]	intracranial	case series	Heparin+ IPC	1277	3.1	2.8	0.3	clinical
Agarwal, 2019 [1]	intracranial/spinal	case series	LMWH+IPC	11436	0.6	0.2	0.4	DUS
Patel, 2013 [34]	intracranial/spinal	case series	IPC + heparin	1277	3.1	2.8	0.3	DUS
Karsy, 2020 [21]	intracranial/spinal	case series	IPC+Heparin/LMWH	1918	22.3	22.3		DUS, D-dimer
Ebeling, 2018 [13]	intracranial	comparative study	CS+LMWH+IPC	75	9.3	8.0	1.3	clinical
Prell, 2018 [35]	intracranial	RCT	CS+LMWH+IPC	41	7.3			DUS
Chibbaro, 2018 [8]	intracranial/spinal	comparative study	CS+LMWH+IPC	3818	1.5	1.3	0.2	DUS
Eisenring, 2013 [13]	intracranial/spinal	comparative study	IPC+LMWH	242	7.1	4.6	2.5	clinical
			CS+LMWH	482	12.7	4.8	7.9	
Ebeling, 2018 [13]	intracranial	comparative study	CS+LMWH	78	6.4	3.8	2.6	clinical
Prell, 2018 [35]	intracranial	RCT	CS+LMWH	53	26.4			DUS
Al-Dujaili, 2012 [2]	spinal	case series	LMWH+CS	158	0.6	0.6		DUS
Carrabba, 2018 [6]	intracranial/spinal	case series	LMWH+CS	275	29.8		2.9	DUS
Chibbaro, 2018 [8]	intracranial/spinal	comparative study	CS+LMWH	3169	4.2	3.0	1.2	DUS
Samuel, 2019 [40]	intracranial/spinal	case series	pharmacologic and mechanical prophylaxis (standard screening)	104	10.6	10.6		clinical
		case series	pharmacologic and mechanical prophylaxis (routine DUS screening)	53	9.4	9.4		DUS
Li, 2020 [25]	intracranial/spinal	case series	mechanical and pharmacologic prophylaxis	204	30.9	30.9		DUS
Yun, 2019 [47]	intracranial/spinal	case series	pharmacologic with/without mechanical prophylaxis	13913	1.8			DUS/CT

*VTE* venous thromboembolism; *DVT* deep venous thrombosis; *PE* pulmonary embolism; *CS* compression stockings; *IPC* intermittent pneumatic compression; *LMWH* Low Molecular weight heparin, *DUS* Duplex ultrasound, *CT* computed tomography

### Pulmonary embolism in neurosurgical patients

Seventeen of the 31 studies reported the incidence of PE in neurosurgical patients (Table 3) [1, 3, 6–10, 12–14, 18, 20, 24, 33, 34, 38, 41].

The reported incidence of PE varied from 0 to 7.9%. A PE incidence of 0 to 4.5% was reported after intracranial surgery, 0.4 to 2.8% after spinal surgery, and 0.2 to 7.9% after intracranial/spinal surgery. The limited amount of data on the incidence of PE after neurosurgical procedures without antithrombotic prophylaxis reported an incidence of 1.0% after intracranial surgery.

Studies that provided pharmacologic prophylaxis report a PE incidence of 0 to 4.5% after intracranial surgery and 0.4% after intracranial/spinal surgery, that of mechanical prophylaxis a PE incidence of 0.5 to 3.4% after intracranial surgery, and that of both pharmacologic and mechanical prophylaxis a PE incidence of 0.3 to 2.6% after intracranial surgery and 0.2 to 7.9% after intracranial/spinal surgery.

### Best evidence synthesis

Our analysis yielded 14 studies with a low risk of bias (Tables 1 and 2, printed in bold; Table 5) [1, 2, 6, 10, 16, 19–21, 25, 30, 34, 35, 40, 41]. In these studies, the reported incidence of VTE was 4.0% (clinical evaluation) in patients not receiving antithrombotic prophylaxis [10]. In patients receiving pharmacological prophylaxis in intracranial/spinal surgery, VTE rate was 31.1% [16] using systematic screening methods and 2.0% [10] using clinical evaluation. In patients who received mechanical prophylaxis, the incidence was 12.9% [41] evaluated on clinical grounds and systematic screening yielded 10.2% [20] or 21.3% [30] in intracranial surgery and 29.4% [19] in spinal surgery. In patients in whom the administration of pharmacological prophylaxis was combined with mechanical prophylaxis, systematic screening yielded VTE rates of 7.3% and 26.4% [35] in intracranial surgery (0.6 [1] to 30.9% [25] in intracranial/spinal surgery), while mere clinical evaluation yielded a percentage of 10.6 [40].

**Table 5:** Incidence of VTE in low-risk bias article categorized by type of operations

Author, Year	Type of operations	Design of studies	Prophylaxis	Sample size	Incidence of VTE (%)	Incidence of DVT (%)	Incidence of PE (%)	Diagnosis of VTE/DVT
Daley, 2015 [10]	intracranial	Case series	LMWH	45	2.0	2.0	0.0	clinical
			no prophylaxis	226	4.0	3.0	1.0	
Nakano, 2018 [30]	intracranial	Case series	CS + IPC	61	21.3			DUS, D-dimer
Shi, 2020 [41]	intracranial	Case series	CS+IPC	1670	13.4	12.9	0.5	clinical
Kaewborisutsakul, 2020 [20]	intracranial	Case series	IPC	177	10.2	8.5	3.4	DUS
Prell, 2018 [35]	intracranial	RCT	CS+LMWH	53	26.4			DUS
			CS+LMWH+IPC	41	7.3			
Al-Dujaili, 2012 [2]	spinal	Case series	LMWH+CS	158	0.6	0.6		DUS
Ikeda, 2017 [19]	spinal	Case series	CS+IPC	194	29.4	29.4		DUS, D-dimer
Guo, 201 5 [16]	intracranial/spinal	Case series	LMWH	196	31.1	31.1		DUS, D-dimer
Agarwal, 2019 [1]	intracranial/spinal	Case series	LMWH+IPC	11436	0.6	0.2	0.4	DUS
Patel, 2013 [34]	intracranial/spinal	Case series	IPC+ heparin	1277	3.1	2.8	0.3	DUS
Carrabba, 2018 [6]	intracranial/spinal	Case series	LMWH+CS	275	29.8		2.9	DUS
Samuel, 2019 [40]	intracranial/spinal	Case series	pharmacologic and mechanical prophylaxis (standard screening)	104	10.6	10.6		clinical
			pharmacologic and mechanical prophylaxis (routine DUS screening)	53	9.4	9.4		DUS
Karsy, 2020 [21]	intracranial/spinal	Case series	IPC+heparin / LMWH	1918	22.3	22.3		DUS, D-dimer
Li, 2020 [25]	intracranial/spinal	Case series	mechanical and pharmacologic prophylaxis	204	30.9	30.9		DUS

*VTE* venous thromboembolism; *DVT* deep venous thrombosis; *PE* pulmonary embolism; *CS* compression stockings; *IPC* intermittent pneumatic compression; *LMWH* Low Molecular weight heparin, *DUS* Duplex ultrasound, *CT* computed tomography

In these studies, the reported incidence of PE varied from 0 [10] to 3.4% [41]. The incidence of PE after neurosurgical procedures without antithrombotic prophylaxes reported an incidence of 1.0% [10]. Studies that provided pharmacologic prophylaxis report a PE incidence of 0% [10] and 0.4% [1], providing mechanical prophylaxis a PE incidence of 0.5 [41] to 3.4% [20] and providing both pharmacologic and mechanical prophylaxis a PE incidence of 0.6% [2], 0.3% [34], and 2.9% [6].

Due to insufficient data, pooling of the data was not deemed meaningful, and only a descriptive analysis was performed.

### Risk factors

Data on risk factors associated with VTE revealed many independent risk factors associated with increased odds of VTE: older age [6, 7, 16, 21, 25, 38], presence of pre- or post-op motor deficit [6, 7, 16, 20, 25, 38], lower Karnofsky Performance Scale score [6, 7], peri-operation treatment with dehydration drugs and fibrin-based sealants [16, 30], increase in postoperative days in intensive care [38], intubated > 24 h/reintubated [38], history of VTE [38], presence of tumor [16], tumor histology [7], hypertension [7, 16, 25], infection [30], DM [20], and increased D-dimers [21, 25] (Table 6). The occurrence of VTE was associated with a longer hospitalization period [21, 25]. Spine surgery is associated with decreased odds of VTE [21]. Gender as a risk factor displayed contradictory results [21, 25].

**Table 6:** Risk factor of VTE based on multivariate analysis

Risk factor	Author, year	Grade	OR/HR of VTE/DVT
Gender	Karsy, 2020 [21]	Female	0.6 (0.5–0.8)
	Li, 2020 [25]	Male	0.17 (0.05–0.57)
Age	Rinaldo, 2019 [38]	Older	1.02 (1.01–1.05)
	Chaichana, 2013 [7]	> 65	1.854 (1. 252–2.745)
		Older	1.033 (1.020–1.046)
	Guo, 2015 [16]	Older	3.356 (1.303–6.643)
	Carrabba, 2018 [6]	> 65	> 1 (*P* = 0.011)
	Li, 2020 [25]	Older	1.03 (1.00–1.08, *P* > 0.05)
	Karsy, 2020 [21]	Older	1.01 (1.006–1.019)
Height	Li, 2020 [25]	Higher	1.01 (0.94–1.08, *P* > 0.05)
Obesity (BMI)	Li, 2020 [25]	Higher	1.10 (0.96–1.26, *P* > 0.05)
Pre- or post-op motor deficit	Rinaldo, 2019 [38]		2.64 (1.43–4.88)
	Chaichana, 2013 [7]		1.854 (1.244–2.763)
	Kaewborisutsakul, 2020 [20]		3.64 (1.17–10.23)
	Guo, 2015 [16]		7.717 (3.390–17.569)
	Carrabba, 2018 [6]	Post-op	> 1
	Li, 2020 [25]	Higher GCS score	0.81 (0.68–0.96)
Karnofsky Performance Scale score	Chaichana, 2013 [7]	< 70	1.721 (1.616–2.549)
		Poor KPS	1.040 (1.026–1.052)
	Carrabba, 2018 [6]	< 80	> 1 (*P* = 0.002)
Surgical category	Karsy, 2020 [21]	Vascular	0.95 (0.66–1.36, *P* > 0.05)
		Spine	0.6 (0.4–0.9)
		Trauma	1.1 (0.8–1.7, *P* > 0.05)
		Tumor	1.6 (1.1–2.4)
		Other	1.6 (1.03–2.33)
Peri-operation treatment	Guo, 2015 [16]	Dehydration drug	1.429 (1.072–3.328)
	Nakano, 2018 [30]	Fibrin-based sealants	2.54 (0.64–10.04, *P* > 0.05)
Post-op ICH day	Rinaldo, 2019 [38]	More	4.35 (1.51–12.55)
Intubated > 24 h/reintubated	Rinaldo, 2019 [38]		3.27 (1.28–8.32)
History of VTE	Rinaldo, 2019 [38]		7.26 (3.24–16.27)
Presence of tumor	Guo, 2015 [16]		6.581 (3.219–24.786)
Tumor histology	Chaichana, 2013 [7]	Glioma (high grade)	1.702 (1.176–2.465)
Hypertension	Chaichana, 2013 [7]		1.785 (1.180–2.699)
	Guo, 2015 [16]		1.229 (1.051–1.538)
	Li, 2020 [25]		2.8
Cardiovascular comorbidities	Carrabba, 2018 [6]		> 1 (*P* > 0.05)
Infection	Nakano, 2018 [30]		12.15 (1.09–134.98)
DM	Kaewborisutsakul, 2020 [20]		4.52 (1.38–14.82)
Laboratory result	Karsy, 2020 [21]	D-dimer ≥ 3.5 µg/mL	1.28 (1.01–1.62)
	Li, 2020 [25]	Postoperative D-dimer	1.22 (1.02–1.47)
		Preoperative D-dimer	1.17 (0.93–1.45, *P* > 0.05)
Length of hospital stay	Karsy, 2020 [21]	Longer	1.02 (1.01–1.03)
	Li, 2020 [25]	Longer	1.03 (0.98–1.09, *P* > 0.05)

### Hemorrhage incidence

Data on the incidence of postoperative hemorrhage can possibly related to LMWH as prophylactic treatment for TE and is therefore relevant with respect to the topic [1–3, 6, 8, 10, 14, 30, 38, 42, 43]. The reported incidence of postoperative hemorrhage ranged from 0 to 9.1% (Table 7). Studies that provided pharmacological prophylaxis report a postoperative hemorrhage incidence varying from 0 to 9.1%, and those providing both pharmacological and mechanical prophylaxis reported a postoperative hemorrhage incidence varying from 0.6 to 6.7%. Only one of the articles providing only mechanical prophylaxis provides data on postoperative hemorrhage and reports an incidence of 1.6% [30].

**Table 7:** Incidence of hemorrhage

Author, year	Type of operations	Study design	Prophylaxis	Sample size	Incidence of hemorrhage (%)
Daley, 2015 [10]	Intracranial	Case series	LMWH	45	0
Sjåvik, 2016 [42]	Intracranial	Case series	LMWH routinely	626	9.1
			LMWH as needed	353	6.5
Smith, 2014 [43]	Intracranial	Case series	2 heparin, 23LMWH	336	1.2
Ali, 2019 [3]	Intracranial	Case series	4 heparin 1–4 days, 4 LMWH 1–2 days, the other no	387	0
Nakano, 2018 [30]	Intracranial	Case series	CS + IPC	61	1.6
Rinaldo, 2019 [38]	Intracranial	Case series	LMWH + IPC	1622	1.8
Al-Dujaili, 2012 [2]	Spinal	Case series	LMWH + CS	158	1.9
Agarwal, 2019 [1]	Intracranial/spinal	Case series	LMWH + IPC	11,436	3.7
Carrabba, 2018 [6]	Intracranial/spinal	Case series	LMWH + CS	275	2.5
Eisenring, 2013 [14]	Intracranial/spinal	Comparative study	CS + LMWH	482	6.7
			IPC + LMWH	242	5.0
Chibbaro, 2018 [8]	Intracranial/spinal	Comparative study	CS + LMWH	3169	0.6
			CS + LMWH + IPC	3818	1.0

## Discussion

The prophylaxis strategies to prevent VTE in neurosurgery vary widely [14, 40, 42]. Moreover, the reported incidence of thromboembolic complications with the applied strategies covers a wide range as well [13, 16, 25, 26, 34, 38]. VTE incidence was reported to be substantially higher if the evaluation was done by a systematic screening method in comparison to a clinical evaluation method [35]. Results on incidence of VTE were grouped by type of operation (longer duration and longer anticipated immobilization), type of prophylaxis, and alleged risk of bias in the articles, in order to provide unequivocal results. We have to conclude though that the data available in literature do not allow drawing more specific conclusions on the effectiveness of prophylactic measures to lower the incidence of VTE than reported in our previous review.

It was assumed that interventions on the spine were less invasive than the intracranial procedures and that reported incidence of VTE would be lower in this group of patients. However, data were scarce to begin with and moreover varied widely. This is presumably due to the difference in nature of the spinal interventions. Even the two articles that reported data on specifically spinal surgery patients yielded very different data: one article reports a 0.6% incidence [2] and the other one a 29.4% incidence [19]. Presumably, the nature of the evaluated spinal interventions is different, but the articles do not elaborate on the specific interventions, making it impossible to draw valuable conclusions.

### Detection of subclinical DVT

The question remains whether it is advisable to perform a postoperative evaluation of (subclinical) presence of DVT. The CHEST Guidelines, the evidence-based clinical practice guidelines concerning thromboembolic prevention developed by the American College of Chest Physicians, only recommends to perform a DUS screening in neurosurgical patients who are at high risk for VTE [15]. High risk for VTE is defined as the presence of a SCI or major head injury, without further specification. In our study, we demonstrated that DVT occurs more often without clinical symptoms (1.3 to 7.9% in patients who received pharmacological prophylaxis, 21.3% in IPC combined with CS, and 7.3 to 26.4% if pharmacologic prophylaxis was combined with CS and/or IPC in intracranial surgery and 0.6 to 30.9% if pharmacologic prophylaxis was combined with CS and/or IPC in intracranial/spinal surgery) than that DVT does lead to the conventional triad of symptoms in the leg (swelling, redness, and pain in the calf) [11, 17]. Subclinical DVT is associated with the formation of PE [29], which is a life-threatening complication. Therefore, systematic detection and prevention of subclinical DVT may be considered essential to prevent the serious complication of PE. In order to avoid performing a DUS for all neurosurgical patients in preparation of surgery, the Caprini Score for risk assessment of venous thromboembolism [5] could be used. The Caprini Score gives four risk groups (low, moderate, high, highest risk), and it can be useful to determine which patients should be preoperatively screened. Nonetheless, high-quality reports evaluating the true VTE incidence in neurosurgical patients are lacking, and thus, a trial to evaluate the subclinical incidence and possible risk factors for the presence of DVT is mandatory in order to decide which patients should be routinely screened postoperatively.

### Risk factors for developing venous thromboembolism

Virchow’s triad classically explains the risk factors for VTE: stasis of blood, endothelial injury, and hypercoagulability [27]. Blood stasis is more likely in patients being subjected to long surgery duration and thus longer immobilization, with paresis/paralysis of the legs and with a poor Karnofsky Performance Scale (KPS) score, and are thus more likely to develop thromboembolic complications. The presence of a malignant tumor, especially higher-grade tumors [7], can interact with the host coagulation system and lead to a hypercoagulable state and thus cause VTE [4]. The risk factors that were evaluated in the articles in this review cover a very wide range and address all these factors. Ideally, these factors should be combined with a “thrombosis risk factor assessment” like the Caprini Score in order to optimize a choice for perioperative prophylactic therapy.

### Postoperative Hemorrhage

Perioperative pharmacological anticoagulant therapy in order to prevent VTE theoretically increases the risk for postoperative hemorrhage. Consequently, the Chest Guideline does not recommend pharmacologic anticoagulant therapy for patients with high hemorrhage risk [15]. Only one of the articles describing the risk on postoperative hemorrhage concerns a prophylactic strategy without pharmacological anticoagulant therapy and gives a percentage of 1.6% [30]. This percentage is in the low range of the hemorrhage percentages demonstrated in the other 11 articles in which hemorrhage is described to range from 0 to 9.1% (Table 7) [1–3, 6, 8, 10, 14, 38, 42, 43]. However, the prevalence of hemorrhage is influenced by several other factors, which are not, or only scarcely, mentioned in the articles, and thus, no meaningful conclusions can be drawn.

### Limitations and external validity

Limitations of this review are the heterogeneity of the type of surgical interventions with varying durations, of diagnostic methods, and of applied antithrombotic prophylaxis therapies. Even more importantly, the pathology of the patients, even within one study, varied. VTE incidence is known to be higher in patients with tumor or trauma, and in some studies, patient populations are mixed, while others are more specifically evaluating patients without trauma or tumor [6, 7, 10, 13, 16, 18, 20, 30, 38, 41–43]. Furthermore, two studies used different kinds of pharmacologic prophylaxis in one group [3, 43], and four studies did not describe the method of prophylaxis precisely [1, 25, 40, 47]. This inconsistency between articles induces selection bias, which makes it inadmissible to draw firm conclusions.

### Clinical implementation

A trial to investigate effectiveness of different thromboembolic prophylactic strategies to prevent VTE should consider type and duration of the surgical intervention and take patient-related risk factors into account. In order to evenly distribute these properties over the groups to be evaluated, a randomized controlled trial setup is the most appropriate. Furthermore, it could be considered to start prophylactic strategy perioperative instead of postoperative, because of the long immobilization and hypercoagulability during the surgical intervention.

## Conclusion

Incidence of VTE cannot be represented by only one percentage, but should be specified with respect to type of intervention, duration of immobilization, and presence of risk factors. Low molecular weight heparin, compression stockings, and intermittent pneumatic compression devices were all evaluated to give reduction in VTE, but with the currently available data, no conclusion can be drawn on generalizing the optimum treatment strategy to lower the incidence of thromboembolic complications. The data on incidence and risk factors however can contribute to optimizing prophylactic regimens in individual patients.

## Data Availability

Not applicable.

## Appendix 1

Table 8

**Table 8:** Search strategy

General search string
(Neurosurgical intervention OR Neurosurgical Procedures OR Neurological surgery) AND (Bleeding OR Thrombo-embolism OR Complication OR Clotting OR problem OR haemorrhage) AND indication OR type of patient OR sample OR Medication OR treatment
PubMed search string
(“Neurosurgical intervention”[All Fields] OR “Neurosurgical procedures”[MESH] OR “Neurological surgery”[All Fields] OR neurosurgical[tiab]) AND (“haemorrhage”[MeSH Terms] OR bleeding[tiab] OR thrombo-embolism OR thrombo-embolism [MESH] OR “blood coagulation”[MeSH Terms] OR Complication OR “Venous Thrombosis”[MESH]) AND (prevalence[tiab] OR incidence[tiab])
Embase search string
Neurosurgery AND “(bleeding OR thromboembolism OR blood clotting).kw. OR neurological Complication OR Vein Thrombosis” AND “prevalence OR incidence”
Cochrane search string
(Neurosurgical intervention OR Neurosurgical procedures OR Neurological surgery OR neurosurgical) AND (haemorrhage OR bleeding OR thrombo-embolism OR thrombo-embolism OR blood coagulation OR Complication OR Venous Thrombosis) AND (prevalence OR incidence)
Searching strategy
((“Neurosurgical intervention”[tw] OR “Neurosurgical interventions”[tw] OR “Neurosurgical procedures”[mesh] OR “Anterior Temporal Lobectomy”[mesh] OR “Brain Tissue Transplantation”[mesh] OR “Cerebral Decortication”[mesh] OR “Craniotomy”[mesh] OR “Hypophysectomy”[mesh] OR “Microvascular Decompression Surgery”[mesh] OR “Nerve Transfer”[mesh] OR “Neuroendoscopy”[mesh] OR “Pallidotomy”[mesh] OR “Psychosurgery”[mesh] OR “Split-Brain Procedure”[mesh] OR “Anterior Temporal Lobectomy”[tw] OR “Anterior Temporal Lobectom*”[tw] OR “Brain Tissue Transplantation”[tw] OR “Brain Tissue Transplant*”[tw] OR “Cerebral Decortication”[tw] OR “Cerebral Decorticat*”[tw] OR “Craniotomy”[tw] OR “Craniotom*”[tw] OR “Decompressive Craniectomy”[tw] OR “Decompressive Craniectom*”[tw] OR “Hemispherectomy”[tw] OR “Hemispherectom*”[tw] OR “Hypophysectomy”[tw] OR “Hypophysectom*”[tw] OR “Microvascular Decompress*”[tw] OR “Microvascular Decompression”[tw] OR “Nerve Transfer”[tw] OR “Nerve Transfer*”[tw] OR “Neuroendoscopy”[tw] OR “Neuroendoscop*”[tw] OR “Pallidotomy”[tw] OR “Pallidotom*”[tw] OR “Psychosurgery”[tw] OR “Psychosurg*”[tw] OR “Split-Brain Procedure”[tw] OR “Trephining”[tw] OR “Trephin*”[tw] OR “Neurological surgery”[tw] OR “neurosurgical”[tw] OR “neurosurgical*”[tw] OR “neurosurgery”[tw] OR “neurosurg*”[tw] OR “Nervous System Diseases/surgery”[Mesh] OR “Nervous System/surgery”[Mesh]) AND (“Hemorrhage”[mesh] OR “bleeding”[tw] OR “Hemorrhage”[tw] OR “Haemorrhage”[tw] OR “Hemorrhag*”[tw] OR “Haemorrhag*”[tw] OR “thrombo-embolism”[tw] OR “thromboembolism”[tw] OR “thrombo-emboli*”[tw] OR “thromboemboli*”[tw] OR “Thromboembolism”[mesh] OR “Pulmonary Embolism”[Mesh] OR “Pulmonary Embolism”[tw] OR “Venous Thrombosis”[mesh] OR “Venous Thrombosis”[tw] OR “Venous Thrombo*”[tw]) AND (“prevalence”[tw] OR “incidence”[tw] OR “Prevalence”[Mesh] OR “epidemiology”[Subheading] OR “Incidence”[Mesh])) AND (“2012/10/04”[PDAT]: “3000/12/31”[PDAT])

## Appendix 2

Table 9

**Table 9:** Newcastle–Ottawa quality assessment scale

Selection
1) Is case definition adequate: and representativeness Is the evaluation of thromboembolic event: Standardized with Doppler or CT: 2 points Standardized with question: check on set event: 1 point Only recorded if the patients spontaneously report an event: 0 points Unknown: 0 points
2) Representativeness of the exposed cohort: CONTROLS (pts without TE complication) Are the patients from the same population Yes: 1 point No: 0 points
3) Definition of the controls: Is the outcome tested at baseline? In case of tumor: do they have Doppler BEFORE the intervention: 1 point If no: 0 points
Comparability
Are there any confounders: No: 1 point Yes: 0 points
Outcome
1) Assessment of outcome Blind assessment of Doppler: 2 points Search in the papers of the patient for the complication (database): 1 point Self-report: 0 points
2) Was follow-up long enough for outcomes to occur a) yes (> 2 days after surgery): 1 point b) no or no information: 0 point
3) Adequacy of follow-up of cohorts Prospective: 1 point Retrospective: 0 points

## Appendix 3

Table 10

**Table 10:** Cochrane risk of bias

A.	1. Was the method of randomization adequate? Yes: 1 point
B.	2. Was the treatment allocation concealed? Yes: 1 point
C. Was knowledge of the allocated interventions adequately prevented during the study?	3. Was the patient blinded to the intervention? Yes: 1 point
	4. Was the care provider blinded to the intervention? Yes: 1 point
	5. Was the outcome assessor blinded to the intervention? Yes: 1 point
D. Were incomplete outcome data adequately addressed?	6. Was the dropout rate described and acceptable? LTFU < 20%: 1 point
	7. Were all randomized participants analyzed in the group to which they were allocated? Intention to treat, yes? 1 point
E.	8. Are reports of the study free of suggestion of selective outcome reporting? Yes: 1 point
F. Other sources of potential bias:	9. Were the groups similar at baseline regarding the most important prognostic indicators? No differences: 1 point
	10. Were co-interventions avoided or similar? Similar: 1 point
	11. Was the compliance acceptable in all groups? Yes: 1 point
	12. Was the timing of the outcome assessment similar in all groups? Yes 1 point
